# Decreased histidine-rich glycoprotein and increased complement C4-B protein levels in follicular fluid predict the IVF outcomes of recurrent spontaneous abortion

**DOI:** 10.1186/s12014-022-09383-9

**Published:** 2022-12-17

**Authors:** Xiaohe Sun, Jiamin Jin, Yin-Li Zhang, Yerong Ma, Songying Zhang, Xiaomei Tong

**Affiliations:** 1 Assisted Reproduction Unit, Department of Obstetrics and Gynecology, Sir Run Run Shaw Hospital, Zhejiang University School of Medicine, No. 3 East Qingchun Road, Hangzhou, 310000 China; 2 Key Laboratory of Reproductive Dysfunction Management of Zhejiang Province, Hangzhou, China

**Keywords:** Recurrent spontaneous abortion, Proteomics, HRG, C4B, Biomarker

## Abstract

**Background:**

Recurrent spontaneous abortion (RSA) is a common and complicated pregnancy-related disease that lacks a suitable biomarker to predict its recrudescence.

**Methods:**

Tandem mass tag (TMT) analysis was conducted to obtain quantitative proteomic profiles in follicular fluid from patients with a history of RSA and from control group. ELISA validation of candidate differentially expressed proteins was conducted in a larger group of patients.

**Results:**

A total of 836 proteins were identified by TMT analysis; 51 were upregulated and 47 were downregulated in follicular fluid from cases of RSA versus control group. Gene Ontology and Kyoto Encyclopedia of Genes and Genomes analysis revealed several important pathways were enriched, involving a dysregulated immunoglobulin Fc receptor signaling pathway and overactivated complement cascade pathways. ELISA validated the differential expression of two proteins, histidine-rich globulin (HRG) and complement C4-B (C4B), which were downregulated and upregulated, respectively, in follicular fluid of patients with RSA. We performed receiver operating characteristic curve analysis of the ELISA results with the outcomes of current IVF cycles as classification variables. The area under the curve results for HRG alone, C4B alone and HRG-C4B combined were 0.785, 0.710 and 0.895, respectively.

**Conclusions:**

TMT analysis identified 98 differentially expressed proteins in follicular fluid from patients with RSA, indicating follicle factors that act as early warning factors for the occurrence of RSA. Among them, HRG and C4B provide candidate markers to predict the clinical outcomes of IVF/ICSI cycles, and the potential for modeling an early detection system for RSA.

**Supplementary Information:**

The online version contains supplementary material available at 10.1186/s12014-022-09383-9.

## Introduction

About 15–25% of clinically recognized pregnancies end with miscarriage, which mostly occurs in early gestation [1]. However, 2–5% of couples experience two or more miscarriages, regarded as recurrent spontaneous abortion (RSA), recurrent miscarriage or recurrent pregnancy loss.

RSA is a complicated pregnancy-related disease. The etiology of RSA includes genetic abnormalities, endocrine disorders, congenital or acquired uterine abnormalities, autoimmune factors, and adverse environmental and psychological factors [2, 3]. However, up to 50% of couples with RSA are diagnosed without an exact etiology [4], hindering accurate and effective treatment. Prospective studies found that the risk of miscarriage arose from approximately 11% amongst nulligravidae to more than 40% after three or more pregnancy losses [5], indicating that the risk of miscarriage increases with the number of previous miscarriages. However, in current clinical practice, there is no suitable biomarker to predict the outcome of an upcoming pregnancy. Therefore, it is important to identify biomarkers and explore the underlying pathology of RSA in affected patients.

The recent development of omics techniques offers new possibilities for biomarker discovery. A few previous studies have explored biomarkers by proteomics using serum samples from patients with RSA. Wu et al. conducted serum biomarker analysis using antibody array technology and ELISA validation for patients with RSA. Their findings demonstrated a significant decrease in IFGBP-rp1/IGFBP-7, dickkopf-related protein 3, receptor for advanced glycation end products and angiopoietin-2 levels in RSA patients [6]. However, biomarker screening in this study was restrictexd to the 1000 proteins in the microarray kit, which may not fully cover the differentially expressed protein profile. Another study using isobaric tags for relative and absolute quantitation and parallel reaction monitoring-based quantitative proteomics also examined serum samples from patients with RSA, and reported important biomarkers, such as CD45, pregnancy-specific beta-1-glycoprotein 1 and peroxiredoxin-2 [7]. However, serum proteins can be variable and easily affected by physical alterations of other systems, including the changing microenvironment of reproductive systems, and marked changes at different phases of the menstrual cycle.

Follicular fluid (FF) is produced in growing follicles and it provides the critical vivo medium for follicle growth and oocyte health [8, 9]. In in vitro fertilization (IVF) or intracytoplasmic sperm injection (ICSI) cycles, oocytes are retrieved through transvaginal ultrasound-guided aspiration, 34–36 h after administration of human chorionic gonadotropin. In this process, the FF of mature oocytes is also collected. Therefore, FF from mature follicles may provide a more stable and representative biological sample for further proteomic and biomarker discovery. Kim et al. first investigated FF proteomics for patients with RSA by 2-dimensional electrophoresis and nano-LC–ESI–MS/MS techniques [10]. After the validation of proteomics results in three RSA and three control FF samples by western blotting analysis, they reported five aberrantly expressed proteins. However, the 2-dimensional electrophoresis and nano-LC–ESI–MS/MS technique adopted in this study has limited sensitivity, and the proteomics results need further validation in a larger sample size.

The technique of proteomic study has developed rapidly since the last decade, quantitative mass spectrometry (MS) nowadays can provide nearly full-scale proteome coverage [11]. However, a major limitation of MS technique is its low-throughput comparisons (only binary or ternary). Labeling approaches using isobaric chemical tags can offer Multiplex quantitation [12]. We collected 6 representative FF samples (three RSA vs. three control group), in order to achieve wider coverage and higher accuracy of our proteomics study.

In the present study, we conducted tandem mass tag (TMT) proteomics to explore the protein expression profile of FF from patients with RSA. We also conducted enzyme linked immunosorbent assay (ELISA) measurements in a larger sample size of patients with RSA to further validate the proteomic results and analyze the predictive value of differentially expressed proteins.

## Materials and methods

### Study subjects and design

The study was approved by the Sir Run Run Shaw Hospital (China) Research and Ethics Committee (approval number: 20200821-31). All patients provided written informed consent before enrollment, and obtained materials and questionnaires were processed anonymously.

Samples of FF were obtained from women undergoing IVF/ICSI-embryo transfer/frozen embryo transfer cycles from 2018 to 2019. RSA was defined when patients experienced two or more previous unexplained spontaneous abortions and did not achieve live birth in the IVF/ICSI cycle of the present study. All of the women with RSA history we recruited for proteomic study aimed to achieve pre-implantation genetic testing (PGT-A) cycle yet they failed in blastulation during embryo culture.

Control group (CON) patients had no history of any pregnancy-related disease and achieved live birth in the IVF/ICSI-embryo transfer/frozen embryo transfer cycle of the present study.

Patients with the following conditions were excluded: (1) genital malformation; (2) parents with an abnormal karyotype; (3) endocrine or metabolic disorders, such as polycystic ovary syndrome; (4) autoimmune diseases; (5) endometriosis or adenomyosis; (6) age < 20 or > 45 years, (6) other major diseases; (7) improper drug use, and history of exposure to chemicals or radiation. In total, FF samples from forty-three patients with RSA and forty-four control group patients were collected, among them, six FF samples (half from patients with RSA and half from Control group patients) were collected for TMT proteomic analysis. Controlled ovarian hyperstimulation using a combination of gonadotropin and gonadotropin-releasing hormone-agonist or -analog was adopted to all patients. Ovarian response was monitored according to serum estradiol (E2) levels and ultrasonography. The FF was collected by transvaginal ultrasound-guided aspiration, 34–36 h after administration of human chorionic gonadotropin (5000 IU or 10 000 IU). The collected samples were aliquoted and stored at − 80 °C.

### Sample preparation from follicular fluid

Most abundant proteins in FF samples were depleted using an Agilent Human 14 multiple Affinity Removal System column following the manufacturer’s protocol. A 10 kDa ultrafiltration tube (Sartorius, Guxhagen, Germany) was used for desalination and concentration of low-abundance components. One volume of SDT buffer (4% SDS, 100 mM dithiothreitol, 150 mM Tris–HCl, pH 8.0) was added, boiled for 15 min and centrifuged at 14 000 × g for 20 min. The supernatant was collected and quantified with a BCA Protein Assay Kit (Bio-Rad, USA).

For filter-aided sample preparation digestion, 200 μg protein of each sample was incorporated into 30 μl SDT buffer. UA buffer (8 M Urea, 150 mM Tris–HCl, pH 8.0) was used to remove the detergent, dithiothreitol and other low-molecular-weight components with repeated ultrafiltration (Microcon units, 10 kDa). Subsequently, 100 μl iodoacetamide (100 mM in UA buffer) was added to block reduced cysteine residues and the samples were incubated for 30 min in darkness. The filters were washed with 100 μl UA buffer three times and then 100 μl of 100 mM triethylammonium bicarbonate buffer twice. After that, the protein suspensions were digested overnight at 37 °C by 4 μg trypsin (Promega) in 40 μl triethylammonium bicarbonate buffer, and the resulting peptides were collected as a filtrate. The peptide content was estimated by UV light spectral density at 280 nm using an extinction coefficient of 1.1 of a 0.1% (g/l) solution that was calculated based on the frequency of tryptophan and tyrosine in vertebrate proteins.

Subsequently, 100 μg peptide mixtures of each sample were labeled using TMT reagent according to the manufacturer’s instructions (Thermo Fisher Scientific, USA). A Pierce high-pH reversed-phase fractionation kit (Thermo Fisher Scientific, USA) was used to fractionate TMT-labeled digest samples into 10 fractions by an increasing acetonitrile step-gradient elution according to instructions.

### Mass spectrometry

For nanoLC-MS/MS analysis, each peptide mixture was loaded onto a reverse-phase trap column (Thermo Scientific Acclaim PepMap100, 100 μm × 2 cm, nanoViper C18) connected to a C18-reverse-phase analytical column (Thermo Scientific Easy Column, 10 cm long, 75 μm inner diameter, 3 μm resin) in 0.1% formic acid and separated with a linear gradient of 84% acetonitrile and 0.1% formic acid at a flow rate of 300 nl/min controlled by IntelliFlow technology. Q Exactive mass spectrometer (Thermo Scientific) coupled to an Easy nLC (Proxeon Biosystems, now Thermo Fisher Scientific) was performed for LC–MS/MS analysis. The mass spectrometer was operated in positive ion mode. The MS data was acquired using a data-dependent top10 method, dynamically choosing the most abundant precursor ions from the survey scan (300–1800 m/z) for higher-energy C-trap dissociation fragmentation. The automatic gain control target was set to 3e6, and maximum inject time to 10 ms. The dynamic exclusion duration was 60 s. Survey scans were acquired at a resolution of 70,000 at 200 m/z and resolution for higher-energy C-trap dissociation spectra was set to 35,000 at 200 m/z (TMT 10-plex), and the isolation width was 2 m/z. Normalized collision energy was 30 eV and the underfill ratio, which specifies the minimum percentage of the target value likely to be reached at maximum fill time, was defined as 0.1%. The instrument was run with peptide recognition mode enabled.

### ELISA

A commercial ELISA assay was used to validate selected differentially expressed proteins, following the manufacture’s protocol. The ELISA kit included histidine-rich glycoprotein (HRG) (SEC534Hu, Cloud-Clone Corp.**,** USA) and human complement C4-B (C4B) (SEB305Hu, Cloud-Clone Corp., USA). The FF was diluted 1:40,000 for HRG and 1:100 for C4B.

### Data analysis

MS/MS spectra were searched using MASCOT engine (Matrix Science, London, UK; version 2.2) embedded into Proteome Discoverer 1.4. Search parameters included trypsin as the protease with up to two missed cleavages, and oxidation of methionine as a dynamic modification. Carbamidomethyl and TMT modification at the N-terminus and lysine residues were regarded as fixed modifications. Peptide were set to ± 20 ppm and fragment mass tolerance were set to ± 20 ppm and 0.1 Da. Peptide identifications were filtered with a 1% false discovery rate threshold at the peptide level. Protein ratios were calculated as the median of only unique peptides of the protein. Differentially abundant proteins were identified with a 1.2-fold change and p value < 0.05. The bioinformation analysis of proteomics data used Gene Ontology (GO; http://www.geneontology.org), Kyoto Encyclopedia of Genes and Genomes (KEGG) pathway (http://www.kegg.jp/ or http://www.genome.jp/kegg/), and STRING (https://www.string-db.org/) databases.

## Results

### Altered expression of proteins in FF from patients with RSA

**Fig. 1:**
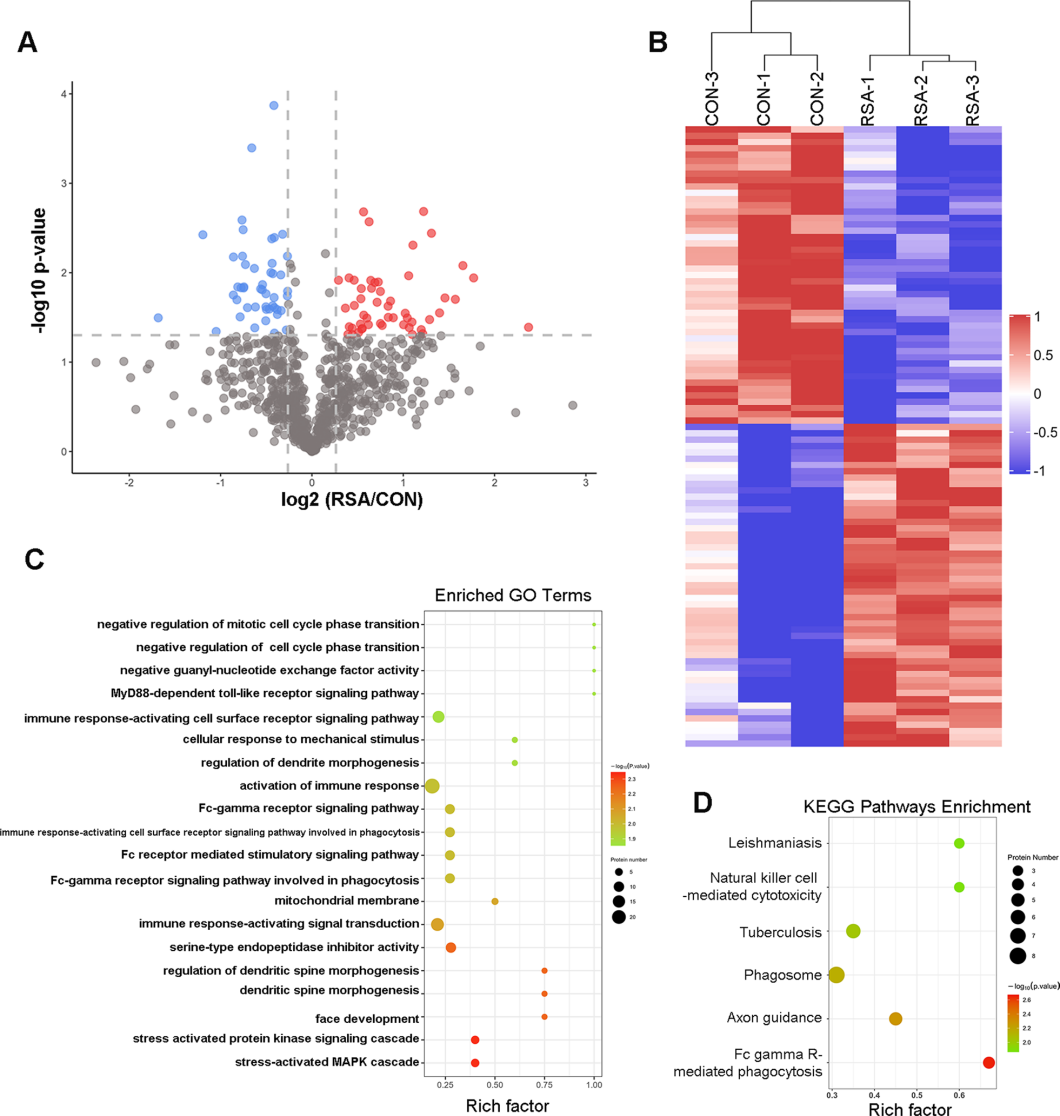
TMT proteomic analysis of follicular fluid samples achieved from RSA and Control group. **A** Volcano plot of the differentially expressed proteins. Blue and Red dots indicate low and high expression level proteins respectively. **B** Heatmap of normalized expression levels of differentially expressed proteins detected from RSA patients (RSA-1, RSA-2, RSA-3) and control group patients (CON-1, CON-2, CON-3). Blue and Red block indicate low and high expression levels respectively. **C** The top 20 enriched GO term: The abscissa in the graph show enrichment to GO function classification and the ordinate represents the number of different proteins under each functional classification; The significance of GO enrichment was calculated by Fisher’s exact test. The p value was indicated by the color of the bar. The numbers on the top of the bar represent the rich factor. **D** Enriched KEGG pathways: The abscissa in the graph represents the number of differentially expressed protein of each KEGG pathways. The significance of KEGG enrichment was calculated by Fisher’s exact test. The p value was indicated by the color of the bar

Six FF samples (three from patients with RSA and three from Control group patients) were collected for TMT proteomic analysis. The clinical parameters of tested samples are shown in Table 1. A total of 836 proteins were identified, among which, 51 were regarded as upregulated and 47 were regarded as downregulated (fold change > 1.2, p value < 0.05, Fig. 1A). The hierarchical clustering of these differentially expressed proteins was visualized in a heat map (Fig. 1B).

**Table 1:** Comparison of TMT analysis participants characteristics between the control group and RSA group (Mean ± SD)

Sample size	Control group	RSA	p-value
	n = 3	n = 3	
Maternal age (y, mean ± SEM)	38 ± 1	37 ± 0.7	ns
BMI	24 ± 0.8	24 ± 1	ns
Number of oocytes retrieved	8 ± 2	7 ± 2	ns
Number of D3 embryo	5 ± 2	3 ± 0	ns
D3/opu	0.6 ± 0.07	0.3 ± 0.3	ns
Number of previous spontaneous abortions (mean ± SEM)	N/A	2.476 ± 0.21	N/A

*y* year, BMI body mass index, *D3* day 3, *opu* oocyte pick-up, *SEM* standard error of mean, *N/A* not available, *ns* not significant

The GO functional annotation and enrichment analysis indicated that the differentially expressed proteins were involved in several important biological processes and molecular functions, such as the stress-activated mitogen-activated protein kinase cascade, stress-activated protein kinase signaling cascades, dendritic spine morphogenesis, serine-type endopeptidase inhibitor activity, bone morphogenetic protein binding, endopeptidase inhibitor activity, endopeptidase regulator activity and peptidase inhibitor activity. Significant changes occurred in cellular components, including the mitochondrial membrane, early endosome membrane, endolysosome, mitochondrial parts and postsynaptic specialization (Fig. 1C).

The KEGG analysis revealed that the most enriched KEGG pathways included immunoglobulin gamma Fc region receptor (FcγR)-mediated phagocytosis, axon guidance, the phagosome, tuberculosis and natural killer cell-mediated cytotoxicity (Fig. 1D).

**Fig. 2:**
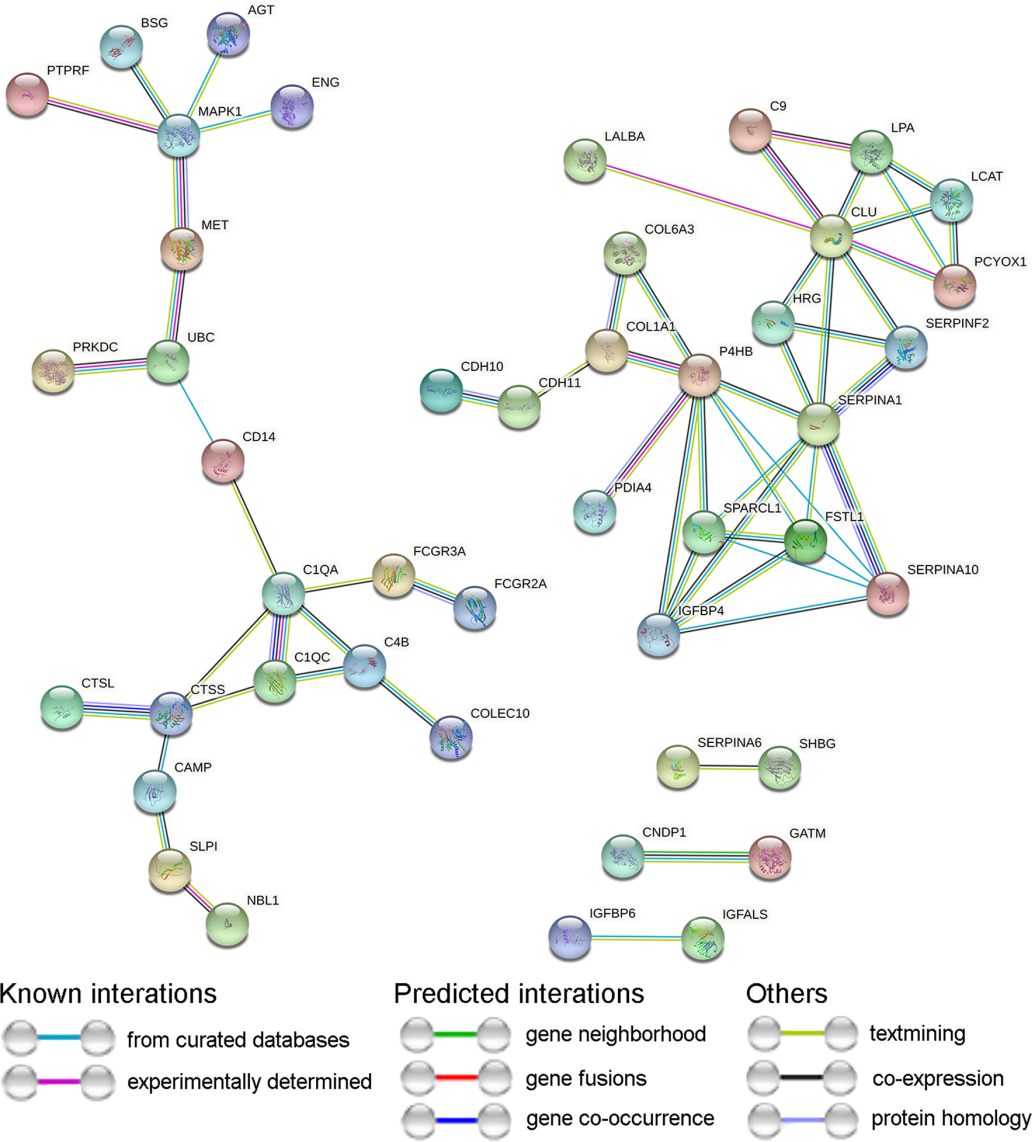
The protein–protein interaction analysis of differentially expressed proteins. The protein–protein interaction network was retrieved form STRING database. The type of interactions was indicated by the line color

Protein–protein interaction analysis for the differentially expressed proteins was retrieved from the STRING database (Fig. 2). The connectivity of a specific protein was regarded as the number of proteins that interacted with it. Components participating in the FcγR-mediated pathways, complement system and mitogen-activated protein kinase cascade had the highest protein connectivity.

**Fig. 3:**
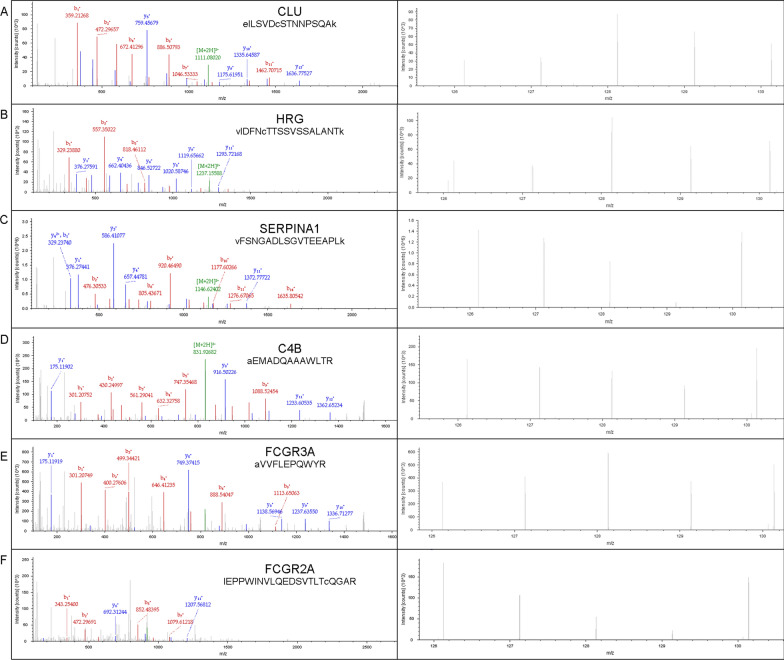
Representive MS/MS spectra of peptides of Clusterin (CLU) (**A**), Histidine-rich glycoprotein (HRG) (**B**), Alpha-1-antitrypsin (SERPINA1) (**C**), Complement C4-B (C4B) (**D**), Low affinity immunoglobulin gamma Fc region receptor III-A (FCGR3A) (**E**) and Low affinity immunoglobulin gamma Fc region receptor II-a (FCGR2A) (**F**). The right column represented the ions labelled by TMT reagent

Representative labeled MS/MS spectra of peptides from differentially expressed proteins [clusterin (CLU), HRG, serpin family A member 1 (SERPINA1), C4B, FCGR3A, FCGR2A] are shown in Fig. 3. The differentially expressed proteins are listed in Table 2.

**Table 2:** Differentially expressed proteins in follicular fluid identified from TMT analysis

Accession	Description	Fold change	*p-*value
P12318	Low affinity immunoglobulin gamma Fc region receptor II-a	5.177551	0.04079
P01817	Immunoglobulin heavy variable 2–5	3.413057	0.011442
A0A075B6K0	Immunoglobulin lambda variable 3–16	3.14558	0.008324
Q92823	Neuronal cell adhesion molecule	2.969312	0.019856
P01706	Immunoglobulin lambda variable 2–11	2.744828	0.019168
P01009	Alpha-1-antitrypsin	2.635182	0.028158
P01877	Immunoglobulin heavy constant alpha 2	2.477888	0.00362
P01782	Immunoglobulin heavy variable 3–9	2.442791	0.033448
P04278	Sex hormone-binding globulin	2.334041	0.002068
A0A0C4DH25	Immunoglobulin kappa variable 3D-20	2.31898	0.048105
Q9NZL9	Methionine adenosyltransferase 2 subunit beta	2.292898	0.043367
Q8TEQ0	Sorting nexin-29	2.152811	0.004921
P06312	Immunoglobulin kappa variable 4–1	2.141848	0.04911
A0A075B6I9	Immunoglobulin lambda variable 7–46	2.136573	0.035698
P78527	DNA-dependent protein kinase catalytic subunit	2.092449	0.041105
A0A0A0MT36	Immunoglobulin kappa variable 6D-21	2.084134	0.010814
A0A0A0MS14	Immunoglobulin heavy variable 1–45	2.0623	0.032063
P49913	Cathelicidin antimicrobial peptide	2.030559	0.02841
P01593	Immunoglobulin kappa variable 1D-33	2.005143	0.038212
P50440	Glycine amidinotransferase, mitochondrial	1.854374	0.031875
P0DP01	Immunoglobulin heavy variable 1–8	1.816197	0.020773
P08519	Apolipoprotein(a)	1.785419	0.031567
P00709	Alpha-lactalbumin	1.782673	0.023685
P35858	Insulin-like growth factor-binding protein complex acid labile subunit	1.704247	0.038891
A0A0B4J1X8	Immunoglobulin heavy variable 3–43	1.682797	0.037027
P01019	Angiotensinogen	1.681592	0.01622
Q86U17	Serpin A11	1.654839	0.012715
P80108	Phosphatidylinositol-glycan-specific phospholipase D	1.640087	0.02142
Q9UK55	Protein Z-dependent protease inhibitor	1.612035	0.012885
Q96KN2	Beta-Ala-His dipeptidase	1.572391	0.014946
P08697	Alpha-2-antiplasmin	1.562821	0.012201
Q86Y38	Xylosyltransferase 1	1.54334	0.002695
P04180	Phosphatidylcholine-sterol acyltransferase	1.535046	0.038166
P08571	Monocyte differentiation antigen CD14	1.517049	0.032188
P02748	Complement component C9	1.48231	0.02984
Q9UHG3	Prenylcysteine oxidase 1	1.479588	0.002089
Q14574	Desmocollin-3	1.466396	0.042045
P13727	Bone marrow proteoglycan	1.457008	0.042756
Q14563	Semaphorin-3A	1.454384	0.015055
Q14956	Transmembrane glycoprotein NMB	1.449926	0.019549
P0C0L5	Complement C4-B	1.445087	0.037566
P02745	Complement C1q subcomponent subunit A	1.425661	0.046568
P08185	Corticosteroid-binding globulin	1.379337	0.023259
P02747	Complement C1q subcomponent subunit C	1.3775	0.012126
Q15166	Serum paraoxonase/lactonase 3	1.358953	0.049062
P35442	Thrombospondin-2	1.357539	0.041683
P12111	Collagen alpha-3(VI) chain	1.32807	0.04021
Q9NZ08	Endoplasmic reticulum aminopeptidase 1	1.325501	0.011439
A0A0B4J2D9	Immunoglobulin kappa variable 1D-13	1.314393	0.049043
Q14624	Inter-alpha-trypsin inhibitor heavy chain H4	1.289411	0.024922
Q9Y6Z7	Collectin-10	1.22374	0.012141
P07237	Protein disulfide-isomerase	0.831644	0.016129
P22692	Insulin-like growth factor-binding protein 4	0.830514	0.006539
P34096	Ribonuclease 4	0.829891	0.018181
P17813	Endoglin	0.823694	0.043789
O14792	Heparan sulfate glucosamine 3-O-sulfotransferase 1	0.800159	0.003716
Q8WWZ8	Oncoprotein-induced transcript 3 protein	0.791685	0.02654
P05451	Lithostathine-1-alpha	0.789616	0.010585
P08581	Hepatocyte growth factor receptor	0.769397	0.029282
Q14515	SPARC-like protein 1	0.768021	0.025812
P49747	Cartilage oligomeric matrix protein	0.752379	0.047458
P10909	Clusterin	0.751895	0.02459
Q9ULJ8	Neurabin-1	0.751698	0.004046
P41222	Prostaglandin-H2 D-isomerase	0.750289	0.018971
P55287	Cadherin-11	0.749237	0.000135
Q92954	Proteoglycan 4	0.743336	0.010246
P10586	Receptor-type tyrosine-protein phosphatase F	0.742341	0.023398
P25774	Cathepsin S	0.739352	0.007871
P24592	Insulin-like growth factor-binding protein 6	0.73734	0.00418
P08637	Low affinity immunoglobulin gamma Fc region receptor III-A	0.732234	0.009967
P29401	Transketolase	0.723494	0.025527
Q96FE7	Phosphoinositide-3-kinase-interacting protein 1	0.707896	0.025723
Q16627	C–C motif chemokine 14	0.705543	0.017215
P54764	Ephrin type-A receptor 4	0.705289	0.024079
P35613	Basigin	0.704373	0.034481
P07711	Cathepsin L1	0.687263	0.013608
P02452	Collagen alpha-1(I) chain	0.685789	0.015527
Q12841	Follistatin-related protein 1	0.677833	0.015196
P13667	Protein disulfide-isomerase A4	0.651188	0.024178
Q6E0U4	Dermokine	0.648035	0.041366
P01889	HLA class I histocompatibility antigen, B-7 alpha chain	0.646108	0.008952
P04196	Histidine-rich glycoprotein	0.633419	0.000402
P41271	Neuroblastoma suppressor of tumorigenicity 1	0.61317	0.024597
Q8TBY8	Polyamine-modulated factor 1-binding protein 1	0.603828	0.008106
P03973	Antileukoproteinase	0.596556	0.014427
P23528	Cofilin-1	0.59567	0.01483
P28482	Mitogen-activated protein kinase 1	0.593313	0.003304
Q6UB99	Ankyrin repeat domain-containing protein 11	0.590385	0.006536
Q9NY97	N-acetyllactosaminide beta-1,3-N-acetylglucosaminyltransferase 2	0.588245	0.002577
Q9Y6N8	Cadherin-10	0.584074	0.014756
Q86VD1	MORC family CW-type zinc finger protein 1	0.579863	0.031196
Q6ZN16	Mitogen-activated protein kinase kinase kinase 15	0.569608	0.014446
Q8NDA8	Maestro heat-like repeat-containing protein family member 1	0.565701	0.020083
Q99650	Oncostatin-M-specific receptor subunit beta	0.551197	0.006674
P30048	Thioredoxin-dependent peroxide reductase, mitochondrial	0.550847	0.017769
P0CG48	Polyubiquitin-C	0.483447	0.045477
Q86SF2	N-acetylgalactosaminyltransferase 7	0.437085	0.003762
Q5SVZ6	Zinc finger MYM-type protein 1	0.311497	0.031967

*fold change* fold change of protein expression level of RSA/control group

### ELISA analysis of differentially expressed proteins in a larger group of patients

To further validate the clinical significance of the identified differentially expressed proteins, we enlarged our sample size by collecting FF samples from 43 patients with RSA and 44 Control group patients. The clinical characteristics and information of IVF cycle were summarized in Table 3.

**Table 3:** Comparison of clinical characteristics of participants in enlarged ELISA analysis between the control group and RSA group

Sample size	Control group	RSA	p-value
	n = 44	n = 43	
Maternal age (y, mean ± SEM)	33 ± 0.6	35 ± 0.8	ns
AMH (ng/mL, mean ± SD)	5 ± 0.5	2 ± 0.2	0.0001
Number of oocytes retrieved (mean ± SEM)	13 ± 0.7	8 ± 0.7	< 0.0001
Number of D3 embryo (mean ± SEM)	7 ± 0.5	4 ± 0.5	< 0.0001
D3/opu (mean ± SEM)	0.6 ± 0.03	0.6 ± 0.05	ns
Number of previous spontaneous abortions (mean ± SEM)	N/A	2.476 ± 0.21	N/A

*y* year, AMH anti-Müllerian hormone, *D3* day 3, *opu* oocyte pick-up, *SEM* standard error of mean, *SD* standard deviation, *N/A* not available, *ns* not significant

ELISA assay was applied for validation experiments. ELISA is a commonly used assay for detecting and quantifying substances. It yields quantitative results and has various proven commercialized kits. Moreover, as a form of body fluid, FF is suitable for ELISA assay.

**Fig. 4:**
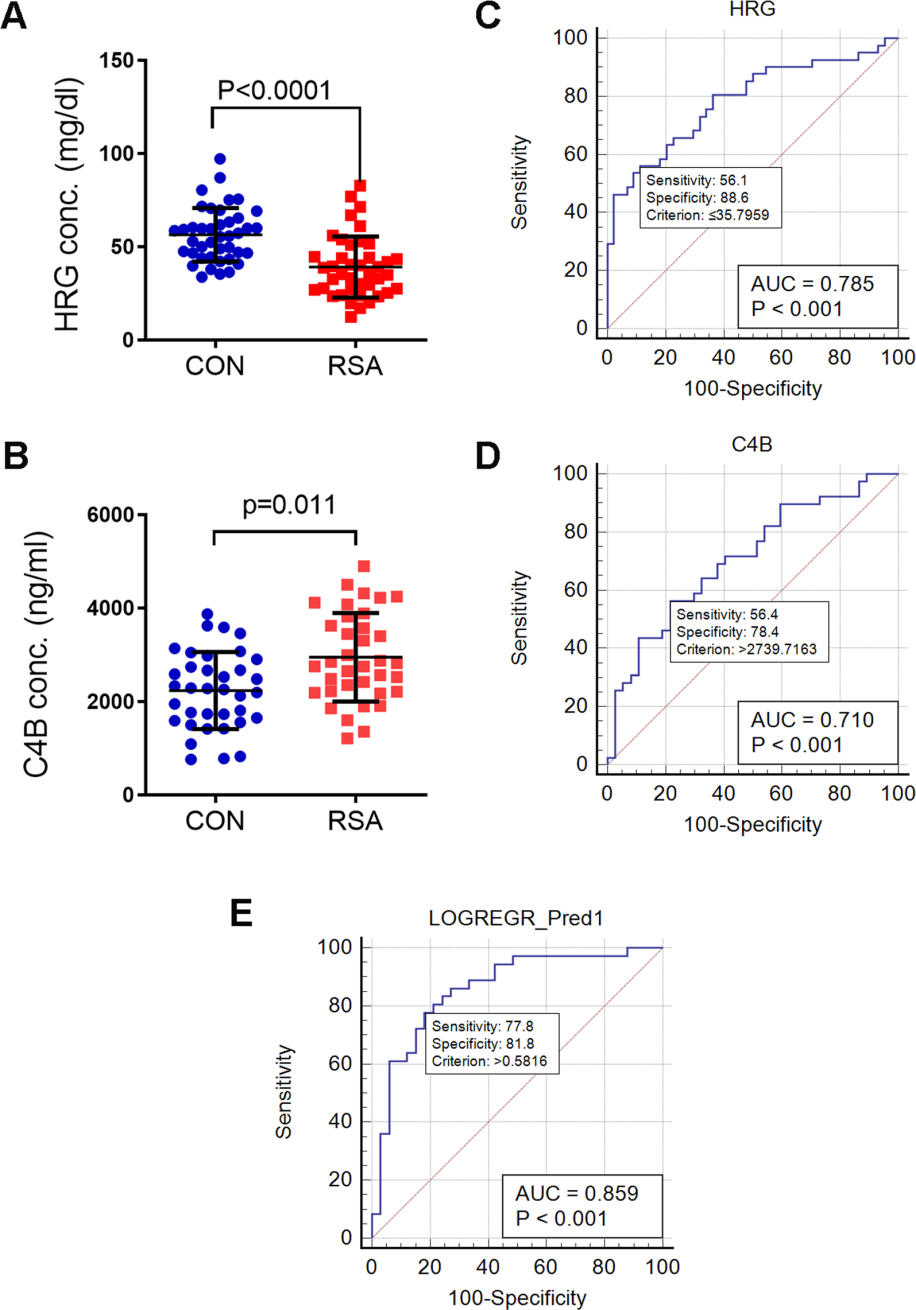
Validation of HRG and C4B expression in enlarged RSA and Control gruop patients. **A** ELISA validation of HRG expression in 41 RSA and 41 Control group patients. **B** ROC analysis of HRG. **C** ELISA validation of C4B expression in 36 RSA and 36 Control group patients. **C**, **D** Receiver operating characteristic (ROC) curves of HRG and C4B respectively. The test variable was protein expression level and the classification variable was the outcome of the current IVF cycle. **E** The ROC curve of HRG and C4B combined, the test variable was the predicted probability calculated by logistic regression of C4B and HRG in the same FF sample. The blue solid line represents the ROC curve. The diagonal line (red dashed line) from [0, 0] to [1, 1] is the line of equivalence. The *p*-value and area under the curve (AUC) was calculated and shown in the lower right corner of each graph

Because of the limited volume of collected FF, some cases were only used for a single protein concentration test. The ELISA results of HRG and C4B expression were consistent with the TMT results (Fig. 4). The concentration of HRG protein was lower in patients with RSA compared with Control group patients (39.15 ± 16.16 versus 56.44 ± 14.42 mg/dl, respectively, p < 0.001) (Fig. 4A). The concentration of C4B protein was higher in patients with RSA compared with CON patients (2948 ± 947.5 versus 2235 ± 822.3 ng/ml, respectively, p = 0.011) (Fig. 4B).

Some of the clinical characteristics and information of IVF cycle are different between the two groups, including the number of oocytes retrieved. Thus we conducted linear regression analysis to identify correlations between clinical parameters, such as age, anti-Mullerian hormone (AMH) levels, number of oocytes, and basal follicle-stimulating hormone levels, and differentially expressed proteins (Table 4). The results of the present study indicated that the expression of HRG and C4B have no correlation with age and number of oocytes retrieved. HRG had a weak positive correlation with AMH (R^2^ = 0.07549, p = 0.0213), while the correlation between C4B and AMH was not significant. Moreover, among patients whose opu number were between five to fifteen, the HRG level was still significantly lower in (54.04 ± 17.07 vs. 39.89 ± 16.89 mg/dl, respectively, p = 0.0029) while C4B level was higher (1994 ± 816.9 vs. 2861 ± 954.0 ng/ml, respectively, p = 0.0045) in RSA group (Additional file 1: Figure). Stratified analysis of HRG and C4B by controlled ovarian hyperstimulation protocols exert no difference among protocols (Additional file 1: Table).

**Table 4:** Correlation between HRG and C4B protein expression level and clinical parameters

Clinical parameter	Liner regression with HRG		Liner regression with C4B	
	R^2^	p-value	R^2^	p-value
Maternal age	0.005313	ns	0.00426	ns
AMH	0.07549	0.0213	0.02352	ns
Number of oocytes retrieved	0.04545	ns	0.01114	ns
D3/opu	0.001758	ns	0.005879	ns

*D3* day 3, AMH anti-Müllerian hormone, *opu* oocyte pick-up, *ns* not significant

### HRG and C4B are candidate markers to predict the outcome of IVF cycles

Receiver operating characteristic curve (ROC) analysis was conducted to evaluate the diagnostic performance of the candidate proteins. For single factor analysis, the level of HRG and C4B in FF samples were regarded as test variables, and outcomes of the present IVF cycle of the patient were regarded as classification variables. The area under the curve (AUC) for HRG was 0.785 (p < 0.001) (Fig. 4C), with a cut-off value < 35.8 mg/dl, test sensitivity of 55% and specificity of 88.6%. The AUC for C4B was 0.710 (p < 0.01) (Fig. 4D), with a cut-off value of > 2739.7 ng/ml, test sensitivity of 56.4% and specificity of 78.4%.

For double-factor analysis, logistic regression of the levels of HRG and C4B in the same FF samples were conducted to calculate the predicted probability. The values of predicted probability were regarded as the test variables, and outcomes of the present IVF cycle of the patient were regarded as classification variables. The AUC for double-factor analysis was 0.859 (p < 0.001) (Fig. 4E), with a test sensitivity of 77.8% and specificity of 81.8%.

## Discussion

THE present study utilized the TMT proteomics technique to explore the FF protein expression profile for patients with RSA. To the best of our knowledge, this is the first study using the TMT technique to identify differentially expressed proteins in FF from RSA samples, providing unique insight into the altered reproductive microenvironment of these patients.

### Dysregulated FcR signaling pathway

The present TMT results revealed upregulation of FcγRII-a and downregulation of FcγRIII-a in FF samples from patients with RSA. Both GO and KEGG analysis indicated significant enrichment of FcγR-mediated pathways, indicating dysregulation of FcγR and its downstream pathways in cases with RSA.

The FcγR was reported to be an important regulator in the maternal–fetal relationship [13, 14]. Like the present study, a recent proteomic study of serum samples from patients with RSA revealed a significant change had occurred in the pathway of FcγR-mediated phagocytosis [7]. These findings indicate that the abnormality of FcγR-related pathways and dysregulation of the immune system in patients with RSA also exist at the level of follicle development.

### Overactivated complement cascade pathways

Complement is a group of proteins that form the core component of the humoral immune system, which protects the host against invading organisms, and initiates inflammation and tissue injury. The complement system is a vital modulator of immune surveillance [15]. A successful pregnancy requires proper immune adaptation of the fetus and placenta, a combination that can be regarded as a semi-allogenic graft. Thus, appropriate complement inhibition is needed to maintain a pregnancy [16, 17], and abnormal activation of complement is reported to be associated with RSA [18, 19]. More than 30 proteins have been recognized as being involved with the complement system [20]. A previous study reported that elevated serum C3 and C4 levels in patients with a history of RSA can be predictors of future miscarriage [21].

In the present study, TMT analysis identified dozens of components of the complement system in FF samples, and several components were found to be significantly upregulated in FF samples from patients with RSA, including C9, C4B, C1QA, and C1QC. Inhibitors of complement activation were downregulated (e.g., CLU), which indicated abnormal complement activation in the microenvironment of follicle growth.

Among the differentially expressed proteins, the peptide-spectrum matches of C4B were the most abundant, indicating it is likely to have higher expression in FF samples. Moreover, C4B is an isotype of native C4 proteins that participates in the classical and mannose-binding lectin complement activation pathways [22]. Therefore, we selected C4B for further study. The results of C4B levels examined in FF samples from a larger sample size of patients were consistent with the TMT analysis, with higher expression levels found in FF samples from patients with RSA compared with control group. ROC analysis of C4B and the reproductive outcomes described above also showed its potential for the prediction of reproductive outcomes; the AUC was 0.710 (p < 0.01). However, a previous proteomics study of serum samples from cases with RSA did not report a significant alteration of components of the complement activation system [7]. We speculate that abnormal component activation is more significant in the follicular microenvironment.

### Low HRG expression levels in FF may be related to several important biological processes

Histidine-rich glycoprotein (HRG) is a 75-kDa single polypeptide, multidomain protein produced by the liver. It can bind to several receptors, including heparin, plasminogen, fibrinogen, and complement components, as well as divalent metal ions [23]. Previous studies revealed the involvement of HRG in coagulation [24], fibrinolysis [25], angiogenesis [26, 27] and the immune system [28, 29].

The present TMT analysis revealed that HRG was significantly downregulated in FF samples from cases with RSA. HRG has been reported to present in the FF, as well as embryos, endometrium, fallopian tube, myometrium, and placenta [30]. Several HRG gene polymorphisms have been reported to be related to the occurrence of RSA [31, 32].

However, the exact role of HRG in the human reproductive system needs further investigation. In the present study, GO analysis of the TMT analysis findings indicated the involvement of HRG in several enriched GO terms, such as serine-type endopeptidase inhibitor activity, endopeptidase inhibitor/activator activity, peptidase inhibitor activity, and positive regulation of immune response.

In addition, HRG has been regarded a regulator of the complement system [23, 29, 33]. It can bind to human C1Q and IgG, and it inhibits the formation of insoluble immune complexes. As mentioned above, C1QA and C1QC were upregulated in the FF samples from patients with RSA. Moreover, HRG has been reported to regulate the expression and function of the FcγR [34, 35]. In summary, HRG has a tight connection with various differentially expressed proteins and pathways, and its downregulation in FF may be an indicator of the dysregulation of various functions, including angiogenesis, coagulation, and the immune system. The correlation between HRG and AMH also revealed its possible relationship with ovarian reserve function.

In the present study, the reduced expression of HRG in FF from patients with RSA was validated by ELISA. Moreover, analysis using ROC curves showed that the expression level of HRG in patients undergoing IVF/ICSI cycles may provide a candidate biomarker to predict reproductive outcomes (e.g., live birth versus not pregnant or miscarriage) in such patients [36]. Consistent with the present study, another study using proteome analysis showed that preconception use of folic acid regulated HRG and downregulated FcγRIII-a in FF samples [37]. Overall, lower HRG expression in ovulating follicles may be an indicator of poor outcomes for future IVF treatment.

However, there are some limitations in our present study: the sample size was relatively small and the unfavorable IVF outcome of RSA patients need to be categorized (no pregnancy; biochemical pregnancy loss, pregnancy loss). Thus, further study is worth pursuing.

In conclusion, the present study identified 98 differentially expressed proteins in the FF of patients with RSA. We also confirmed the abnormal expression of representative proteins, HRG and C4B, in a larger group of patients. Further ROC analysis raised the possibility that HRG protein levels in FF may be used to predict IVF outcomes. This study has provided potential biomarkers to predict the occurrence of RSA, and also demonstrated an abnormal immune system in the follicles of patients with RSA.

## Supplementary Information

**Additional file 1.** Supplementary tables and figure of this research.

## Data Availability

The TMT raw data of this manuscript has been uploaded in Integrated Proteome Resources (https://www.iprox.cn/page/HMV006.html) with the dataset identifier IPX0003868000.
