# Supplementary material for: Decreased histidine-rich glycoprotein and increased complement C4-B protein levels in follicular fluid predict the IVF outcomes of recurrent spontaneous abortion
Source: Clin Proteomics. 2022 Dec 17;19:47. doi: 10.1186/s12014-022-09383-9 (PMC9758815; doi:10.1186/s12014-022-09383-9)
Supplement: Supplementary file 1 — Additional file 1. Supplementary tables and figure of this research. [file 12014_2022_9383_MOESM1_ESM.docx]

**Supplementary Table1** Comparison of clinical characteristics of participants in enlarged ELISA analysis (opu number: 5 to 15)

|  | **CON** | **RSA** | **p-value** |  |
| --- | --- | --- | --- | --- |
| Sample size | n=33 | n=28 |  |  |
| Maternal age (y,mean ± SEM) | 32 ± 0.8 | 35 ± 1 | ns |  |
|  |  |  |  |  |
| AMH (ng/mL, mean±SD) | 3.8 ± 2.1 | 2.8 ± 1.4 | ns |  |
| Number of oocytes retrieved (mean ± SEM) | 9 ± 0.5 | 8 ± 0.5 | ns |  |
| Number of D3 embryo (mean ± SEM) | 6 ± 0.4 | 5 ± 0.4 | ns |  |
| D3/opu(mean ± SEM) | 0.66 ± 0.04 | 0.64± 0.05 | ns |  |

Abbreviations: y, year; D3, day 3; opu, oocyte pick-up; SEM, standard error of mean; SD, standard deviation; N/A, not available; ns, not significant.


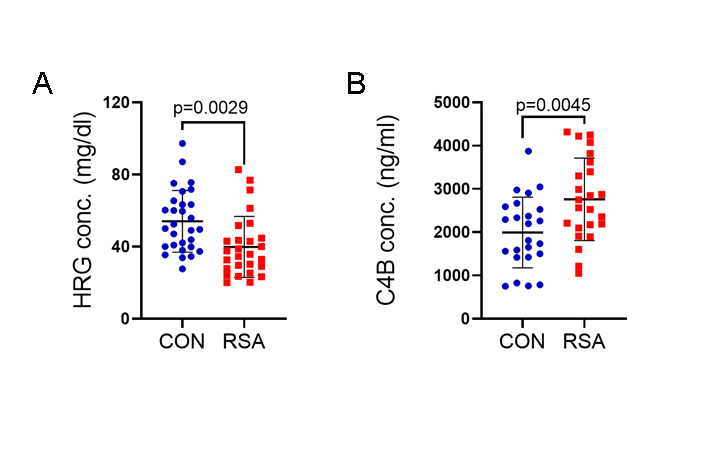


**Supplementary Figure：**HRG (A) and C4B (B) expression in enlarged RSA and CON (patients (opu number between five to fifteen)

**Supplementary Table2:** The expression of HRT and C4B in different controlled ovarian hyperstimulation protocols.

| **Controlled ovarian hyperstimulation protocol** | HRG | | C4B | |
| --- | --- | --- | --- | --- |
|  | **n** | **Mean ± SD (mg/dL)** | **n** | **Mean ± SD (ng/mL)** |
| GnRH-A | 6 | 46.36±9.74 | 6 | 2593±1013 |
| PPOS | 31 | 47.74±22.34 | 31 | 2764±1160 |
| Mild | 10^#^ | 37.18±17.95 | 11^#^ | 3836±2987 |
| EFLL | 5 | 44.67±19.02 | 5 | 2898±544 |
| Long | 36^#^ | 47.60±13.93 | 31^#^ | 2518±1202 |
| ANOVA results | P=0.556 | | P=0.18 | |

#: Due to the limited volume of collected FF, some cases were only used for a single protein concentration test.

Abbreviations: GnRH-A, GnRH-Antagonists protocol; PPOS, progestin primed ovarian stimulation; Mild, mild ovarian stimulation protocol; EFLL, early follicular-phase long-acting GnRH-agonist long protocol
